# Role of CD14 in host protection against infections and in metabolism regulation

**DOI:** 10.3389/fcimb.2013.00032

**Published:** 2013-07-24

**Authors:** Ivan Zanoni, Francesca Granucci

**Affiliations:** Department of Biotechnology and Biosciences, University of Milano-BicoccaMilan, Italy

**Keywords:** pattern recognition receptors, CD14, lipopolysaccharide, NFAT, innate immunity

## Abstract

CD14 is a glycosylphosphatidylinositol (GPI)-anchored receptor known to serve as a co-receptor for several Toll-like Receptors (TLRs) both at the cell surface and in the endosomal compartment. CD14 can be expressed by cells of both hematopoietic and non-hematopoietic origin as a cell membrane or secreted protein. Although CD14 was discovered more than 20 years ago, its activities remain largely to be defined. Most of the information available concerns CD14's role as a co-receptor working with TLR4 and facilitating cellular responses to low doses of lipopolysaccharide (LPS). Recent studies have highlighted and molecularly defined many other functions of this pattern recognition receptor (PRR). These functions include the mechanisms through which CD14 allows the activation of the TLR4-TRAM-TRIF pathway upon LPS stimulation; the capacity of CD14 to transduce a TLR4-independent signaling pathway leading to the activation of NFAT transcription factor family members with important consequences in myeloid cells; the CD14 influence on cell metabolism in conditions predisposing to obesity. In this review, we summarize recent progresses toward the molecular definition of the multiple roles exerted by CD14 in innate immune cells in response to LPS and the consequences of CD14 activation in physiologic and pathologic conditions.

## Introduction

The innate immune system has evolved to sense and respond to a wide range of microorganisms. Microbial detection is mediated by specialized receptors called pattern-recognition receptors (PRRs), which are able to recognize microbe-associated molecular patterns (PAMPs).

PRRs are widely expressed on cells that serve as sentinels of the immune system such as dendritic cells (DCs) and macrophages. After PAMP recognition, PRRs transduce a signal inside the cells leading to transcription factor activation. Activated transcription factors contribute to the initiation of the inflammatory process and induce the maturation of antigen-presenting cells (APCs) involving the upregulation of cell surface and soluble molecules. Thanks to the maturation process, APCs acquire the ability to activate adaptive immunity (Joffre et al., 2009).

Toll-like receptor 4 (TLR4) was the first PRR discovered to be expressed by mammalian innate immune cells (Medzhitov and Janeway, 1997; Medzhitov et al., 1997). After TLR4, 13 new TLRs have been discovered together with additional classes of PRRs, including CLRs (C-type lectin-like receptors), RLRs (RIG-I-like receptors), and NLRs (NOD-like receptors) (Kawai and Akira, 2011).

TLRs are among the best characterized PRRs and are key regulators of anti-bacterial and anti-viral immune responses (Janeway and Medzhitov, 2002). After activation, different adaptor proteins containing the Toll-interleukin (IL)-1 receptor (TIR) domain are recruited to bind to the TIR domains of TLRs. There are four main known adaptor proteins that interact with TLRs: MyD88, TIRAP, TRAM, and TRIF. Through these adaptors, TLRs can activate downstream kinases, including IL-1 receptor-associated kinases (IRAK) (Kawagoe et al., 2007, 2008), mitogen-activated protein (MAP) kinases, and a group of E3 ubiquitin ligases, TRAF (Hacker et al., 2006). The result of this process is a complex regulation of gene transcription mediated by the activation of transcription factors such as NF-kB, AP-1, and IRFs (Medzhitov and Horng, 2009).

Before the discovery of TLR4, CD14—first identified as a marker of monocytes (Goyert et al., 1986)—was proposed to be a PRR (Pugin et al., 1994). It was thought to signal intracellular responses after the recognition of a vast array of bacterial products, including lipopolysaccharide (LPS) (Pugin et al., 1994). CD14 is a 55 kDa glycoprotein expressed on the surface of myelomonocytic cells as a glycosylphosphatidylinositol (GPI)-anchored receptor or can be secreted in a soluble form (Ulevitch and Tobias, 1995). It seemed, therefore, improbable it could have signaling capacities given the absence of an intracellular tail. For a long time, efforts have concentrated on defining its role as the TLR4 and TLR1/2 co-receptor and more recently as the co-receptor of other TLRs, including TLR3, 6, 7, and 9 (Akashi-Takamura and Miyake, 2008; Baumann et al., 2010; Weber et al., 2012).

Recent advances in CD14 biology have confirmed its role not only as the TLR co-receptor but also as a PRR (Zanoni et al., 2009, 2011). Moreover, in addition to the functions in innate immunity (Ostuni et al., 2010), a more general role for CD14 in regulating metabolism, insulin resistance, and obesity is emerging (Johnson et al., 2004; Roncon-Albuquerque et al., 2008; Fernandez-Real et al., 2011). The purpose of this mini-review is to give an overview of the recent progress on the definition of the multiple roles exerted by CD14 in response to LPS and microbial infections. The influence of this PRR on cell metabolism in conditions predisposing to obesity will also be considered.

## CD14 and the LPS receptor complex

LPS is the major component of the outer membrane of Gram-negative bacteria. LPS consists of lipid A, a core polysaccharide, and an O-polysaccharide chain of variable length (often more than 50 monosaccharide units). Colony morphology (“smooth” vs. “rough”) is indicative of the O-glycosylation status. Microbial variants with long O-polysaccharide chains form smooth colonies, whereas those that have short, truncated, O-polysaccharide chains form rough colonies (Kelly et al., 1991).

CD14, together with TLR4 and MD-2, forms the multi-receptor complex that recognizes LPS on the cell membrane (Miyake et al., 2000; Triantafilou and Triantafilou, 2002). The structures of mouse and human CD14 have been crystallographically solved. Human and mouse CD14 are very similar and show the horseshoe-like structure typical of leucine-rich-repeat-containing proteins with a hydrophobic pocket at the NH2-terminal side. This pocket has a cluster of positively charged residues at the rim that presumably accommodates acylated ligands like the phosphorylated lipid A moiety (Kim et al., 2005; Kelley et al., 2013). However, while mouse CD14 crystallizes as a dimer only, human CD14 crystallizes as a monomer. Compared with mouse CD14, human CD14 contains an expanded N-terminal pocket and different rim residues that are likely to be important for ligand binding and cell activation, although the significance of these differences remains to be determined (Kelley et al., 2013). Moreover, since CD14 can also bind the carbohydrate portion of LPS (Pugin et al., 1994), it has been proposed that additional hydrophilic regions spanning outside the N-terminal pocket but in close proximity to it can contribute to LPS binding (Kim et al., 2005). This flexible CD14 structure could explain why CD14 is capable of binding different LPS species with similar affinity and also, perhaps, the promiscuity of this PRR, which serves as a co-receptor for TLR1, 2, 3, 4, 6, 7, and 9, contributing to ligand recognition (Akashi-Takamura and Miyake, 2008; Baumann et al., 2010; Weber et al., 2012).

The first role described for CD14 in LPS recognition was the enhancement of the sensitivity of innate immune cells to this inflammatory stimulus. CD14 is capable of binding LPS at picomolar concentrations and presenting and transferring it to the TLR4-MD2 complex for the initiation of the transduction pathway (Gioannini et al., 2004). CD14-deficient macrophages display a markedly reduced sensitivity to low concentrations of LPS compared to wild-type cells (Perera et al., 1997). Moreover, CD14-deficient mice do not develop septic shock after LPS or Gram-negative bacterial exposure, while wild-type mice do (Haziot et al., 1996).

TLR4 is unique because it engages all four adaptors—TIRAP, MyD88, TRAM and TRIF—and thus is the only TLR capable of activating both the TIRAP-MyD88-dependent pathway and the TRAM-TRIF-dependent pathway leading to the secretion of type-I interferons (IFNs) (Akira and Hoshino, 2003).

In the presence of LPS, CD14 and TLR4-MD2 are brought in close proximity (da Silva Correia et al., 2001) inside lipid rafts (Schmitz and Orso, 2002). After translocation into the lipid rafts, TLR4 in the plasma membrane activates the TIRAP-MyD88-dependent pathway, which leads to the first wave of NF-kB activation (Kawai et al., 2001). Subsequently, the entire receptor complex, including CD14, is internalized (Zanoni et al., 2011) and redirected to the endosomal compartment. The activation of the TRAM-TRIF pathway starts from the endosome (Kagan et al., 2008).

Jiang and colleagues (Jiang et al., 2005) have demonstrated that CD14 is absolutely required for LPS-induced activation of the TLR4/TRAM-TRIF pathway even at very high LPS doses, when CD14 is dispensable for LPS recognition by the TLR4-MD2 complex. In the absence of CD14, both smooth and rough LPS cannot induce TRAM-TRIF-dependent IRF3 activation and subsequent type-I IFN production.

**Figure 1:**
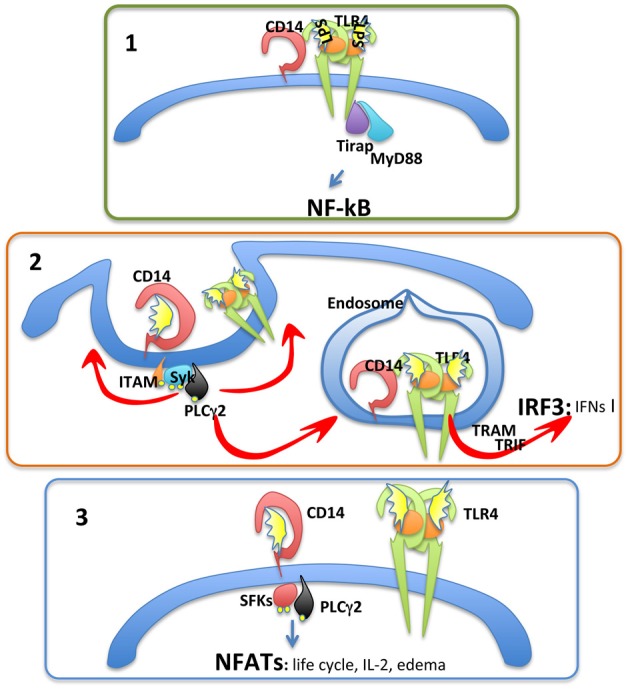
**CD14 has three fundamental functions**. **Panel 1**, CD14 favors optimal NF-kB-dependent cytokine production by allowing the recognition of low-LPS doses. **Panel 2**, CD14 promotes TLR4 endocytosis and type-I IFN expression. **Panel 3**, CD14 has autonomous signaling functions leading to the activation of NFATc transcription factor family members. The consequences of NFAT activation are mostly unknown.

We have recently found that CD14 is capable of controlling the entire LPS receptor complex endosomal re-localization, therefore explaining why type-I IFN production is CD14 dependent (Zanoni et al., 2011). ITAM-bearing molecules, the tyrosine kinase Syk, and its downstream effector phospholipase Cγ 2 (PLCγ 2) have also been identified as important regulators of the CD14-mediated TLR4 endosomal re-localization (Figure 1) (Zanoni et al., 2011). CD14 is dispensable for type-I IFN production only if LPS can directly reach the endosomal compartment through an endocytic process independent of TLR4 recognition, for instance when it is administered with liposome combinations (Watanabe et al., 2013) or associated with microbeads (Zanoni et al., 2011).

Based on these observations it emerges that CD14 plays a crucial role in regulating cellular responses to LPS by: (i) controlling LPS presentation to TLR4 with a consequent facilitation of cellular responses to low LPS doses; and (ii) controlling the re-localization of the LPS receptor complex to the endosome, irrespective of the LPS concentration, with the consequent activation of the TRAM-TRIF pathway and type-I IFN production. These two pathways are depicted in Figure 1.

## CD14 signaling capacities

In addition to controlling the TRIF-dependent pathway, CD14 has TLR4-independent signal transduction capacities in myeloid cells, such as DCs (Zanoni et al., 2009). Following LPS stimulation, CD14 leads to src family kinase (SFK) and PLCγ 2 activation. PLCγ 2, in turn, hydrolyzes membrane phosphatidylinositol bisphosphate (PIP2) generating inositol-triphosphate (IP3) and dyacylglycerol (DAG) (Zanoni et al., 2009). This leads to an increase in intracellular Ca^2+^ concentration and the activation of the phosphatase Calcineurin. Cytoplasmic NFAT transcription factors are, consequently, dephosphorylated and can migrate to the nucleus (Zanoni et al., 2009). This process is depicted in Figure 1.

It remains unclear how CD14 can transduce this signaling pathway and, in particular, whether CD14 induces Ca^2+^ fluxes directly or indirectly. There are two possibilities: (i) CD14 directly induces Ca^2+^ mobilization by recruiting SFKs at the lipid rafts and inducing their activation; or (ii) CD14 presents LPS to an unknown protein (by analogy with LPS presentation to TLR4), which in turn activates the calcium pathway. Although the second hypothesis cannot be formally excluded, indirect evidence favors the first one. Moreover, a direct role in Ca^2+^ mobilization through interactions with lipid rafts and SFK activation has been demonstrated for other GPI-anchored receptors, such as CD59 (Suzuki et al., 2007a,b). Understanding these details of the CD14 signaling capacity would contribute to answering, at least in part, the old question of how GPI-anchored receptors initiate a signaling pathway.

The NFATc proteins were originally identified as transcription factors capable of regulating IL-2 production in T cells (Shaw et al., 1988). Since then, different NFATc functions in adaptive immunity have been identified, including roles in T- and B-cell differentiation and activation (Wu et al., 2007). In innate immune cells, little is known about the NFATc family role, although there is substantial evidence that the NFATc signaling pathway can be activated in physiological and pathological conditions (Zanoni and Granucci, 2012).

At the moment, few but important biological processes regulated by the CD14-NFAT pathway have been described in LPS-activated DCs and include regulation of IL-2 (Granucci et al., 2001) and prostaglandin (PG) E_2_ (PGE_2_) (Zanoni et al., 2012) production and the induction of a proapoptotic pathway in terminally differentiated DCs (Goodridge et al., 2007; Zanoni et al., 2009).

In the context of LPS-mediated inflammatory conditions, DC-derived IL-2, produced in a CD14-dependent manner, is one of the cytokines required, at least in the mouse system, to elicit IFN-γ production from NK cells (Granucci et al., 2004). IFN-γ potently activates macrophages and favors Th1-lineage commitment of CD4^+^ T cells. As such, it comes as no surprise that early IFN-γ release by NK cells is crucial for controlling a variety of bacterial infections, including *E. coli*, while adaptive immunity is being primed (Dunn and North, 1991; Ferlazzo et al., 2003; Newman and Riley, 2007; Lapaque et al., 2009; Pontiroli et al., 2012).

The role of PGE_2_ produced in a CD14-dependent manner during LPS-mediated inflammatory conditions has also been widely investigated. Prostanoids, such as PGE_2_, contribute to the generation of local edema formation by controlling vasodilation (Legler et al., 2010). It has emerged that DCs are the major regulators of edema formation induced *in vivo* by LPS administration, and they exert this function thanks to their capacity to activate the CD14-NFAT pathway (Zanoni et al., 2012). In particular, the NFATc transcription factor family controls the transcription of *Ptges1* that encodes a protein called microsomal PGE synthase 1 (mPGES-1). mPGES-1, together with phospholipase A2 (cPLA2α) and cyclooxygenase-2 (COX-2), regulates PGE_2_ synthesis and release. PGE_2_ in turn favors local swelling formation (Zanoni and Granucci, 2012).

## Role of CD14 in host defense

The role of CD14 during microbial infections remains largely to be defined, despite the progress in the definition of CD14 functions as a TLR co-receptor and PRR.

The CD14 involvement in host defense against viral and bacterial infections has been investigated in several experimental models with opposing results. An essential protective role of CD14 has been established in some forms of intestinal infections, while positive and negative effects have been described in pulmonary infections depending on the infectious agent. A globally detrimental role for CD14 has, finally, been shown in systemic infections.

CD14 has proved to be required for host defense in two models of Gram-negative, clinically relevant, respiratory pathogens, i.e., *Haemophilus influenzae* and *Acinectobacter baumanii* (Wieland et al., 2005; Knapp et al., 2006). In both cases, CD14 was required for bacterial clearance but not for the establishment of the inflammatory process that required, instead, TLR4. Analogously, CD14 has proved to be essential for bacterial clearance in a rabbit model of *E. coli*-induced pneumonia. Also in this case, no effect on neutrophil infiltration and cytokine production has been described in CD14-deficient conditions (Frevert et al., 2000). A detrimental role of CD14 has been defined in the responses against *Streptococcus pneumoniae* (Dessing et al., 2007), a Gram-positive bacterium causing pneumonia, and *Burkholederia pseudomallei*, a Gram-negative bacterium causing melioidosis (Wiersinga et al., 2008). CD14-deficient mice, unlike wild-type mice, were protected from severe lung inflammation, bacterial outgrowth, and sepsis induced by intranasal bacterial instillation (Wiersinga et al., 2008).

In models of gut infection, rabbits treated with a neutralizing anti-CD14 antibody exhibited a remarkably higher susceptibility to infections with *Shigella*, the cause of bacillary dysentery (Wenneras et al., 2001). More severe tissue damage and a strong increase in bacterial intestinal mucosa invasion were observed in CD14-impaired rabbits, while the local inflammatory process was similar in antibody-treated and untreated animals.

Concerning systemic infections, CD14-deficient mice were resistant to septic shock induced by intraperitoneal or endovenous *E. coli* administration (Haziot et al., 1996), and showed a much better control of bacterial spread compared to wild-type animals. Further studies have demonstrated that timing and efficiency of peritoneal neutrophil recruitment in mutant animals accounted for reduced bacteremia (Haziot et al., 2001).

In conclusion, the contribution of CD14 to infection control may be either positive or negative depending both on the microorganism and the site of infection. The regional influence exerted by CD14 would support the hypothesis proposed some years ago that CD14 expression by non-hematopoietic cells could influence innate immune functions (Jersmann, 2005). It would be interesting to understand the functions of CD14 in non-hematopoietic cells to better appreciate its regulatory activities in re-establishing homeostatic conditions.

## Role of CD14 in metabolism

In addition to playing a role in host defense, PRRs have been recently appreciated for their role in metabolism regulation (Konner and Bruning, 2011; Tannahill and O'Neill, 2011). Although, at the moment, the connection between these two functions is only partially understood (Tannahill and O'Neill, 2011), a link could be represented by the general necessity to restore homeostasis after tissue damage caused either by infection or obesity, or various kinds of endogenous signals.

Concerning CD14, an intriguingly negative contribution in the regulation of the body plan has been observed (Johnson et al., 2004). CD14-deficient mice have an ideal body plan with decreased body fat and increased mineral content in the bones compared to wild-type mice. These differences are intensified with age with the consequence that mice have an increased lifespan in the absence of CD14, independent of the genetic background. Accordingly, CD14-deficient mice do not become obese on a high-fat diet and do not develop obesity-related pathologies such as insulin resistance and cardiovascular complications (Roncon-Albuquerque et al., 2008); these phenomena are CD14-dependent but TLR4-independent. On a high-fat diet, CD14 expressed on both hematopoietic cells and adipocytes contributes to mesenteric fat accumulation and type-2 diabetes development (Fernandez-Real et al., 2011).

The mechanism through which CD14 participates in the regulation of lipogenesis, adipose tissue inflammation, and insulin resistance is not known. One possibility is that unknown endogenous ligands activate a CD14-dependent pathway leading to the production of key factors for lipid accumulation. Otherwise, CD14 may influence the intestinal flora composition with a consequent effect on food absorbance and weight gain. This is an attractive hypothesis. High amounts of soluble CD14 used to compete with membrane CD14 were proven to be effective in reducing adipocyte-mediated inflammation and insulin resistance but ineffective in reducing body weight (Fernandez-Real et al., 2011). Therefore, only the obesity-associated pathologies could be corrected by soluble CD14 but not obesity itself. This is consistent with the hypothesis that, if the intestinal flora is altered in CD14-deficient mice, an acute treatment with soluble CD14 could not restore the intestinal flora composition. Another interesting possibility is that LPS could be the cause of CD14 activation, lipogenesis, obesity-driven inflammation, and insulin resistance. This last hypothesis is presumably the most probable one. Although there is no direct evidence that LPS can induce obesity via the activation of a CD14-dependent pathway, indirect observations strongly support this assumption: (i) in humans, triglyceride-rich lipoproteins contain LPS that in normal conditions is probably efficiently sequestered by soluble CD14; (ii) LPS serum levels largely increase in high-fat diet; and (iii) increased LPS levels are sufficient *per se* to predispose to obesity, insulin resistance, and type-2 diabetes (Cani et al., 2007). Interestingly, NFATc2/c4-deficient mice are resistant to high-fat-diet-induced obesity, indicating that the activation of the NFAT signaling pathway is absolutely required for fat accumulation (Yang et al., 2006). Given the capacity of CD14 to initiate the NFAT pathway, this observation calls for an evaluation of the role of the CD14/NFAT signaling pathway in the control of obesity and insulin resistance once induced by LPS in hematopoietic cells and/or in non-hematopoietic cells including adipocytes. It would be interesting to understand whether the CD14-initiated NFAT signaling pathway regulates the production of key factors controlling lipid accumulation.

## Concluding remarks

CD14, one of the first identified PRRs, plays multiple roles in microbial recognition and signaling. It helps recognition of ligands for TLR1, 2, 3, 4, 6, 7, and 9, and it contributes in at least three different ways to the initiation of the signaling pathways activated in response to LPS: CD14 facilitates LPS recognition and the initiation of the MyD88-dependent pathway from the cell membrane; it is required for the LPS receptor complex re-localization in the endosome and the activation of the TRIF-dependent pathway; it starts the calcium/NFAT pathway.

While it is indubitable that CD14 participates in the activation of multiple signaling pathways in response to microbial stimuli, its role in host defense is not always protective, as previously outlined. Moreover, a curious negative role of CD14 in lipid accumulation, obesity, insulin resistance, and type-2 diabetes has been documented. Nevertheless, from an evolutionary point of view, although adipocyte differentiation and lipid accumulation with high efficiency may be detrimental in conditions of high food availability, they could represent an important survival advantage in conditions of limited alimentary resources. Therefore, the real evolutionary advantage provided by CD14 could have been related not only to innate immunity and the protection against specific infectious agents but also to metabolism by facilitating the generation of energy stores.

Many fundamental questions remain unsolved. These questions include: (i) how CD14 initiates the NFAT signaling pathway and mediates the endosomal TLR4 re-localization in response to LPS; (ii) how CD14 contributes to host protection or, *vice-versa*, is deleterious depending on the type of infection; (iii) how CD14 and possibly the CD14/NFAT pathway regulate metabolism and lipid accumulation; (iv) what is the role of CD14 expressed by non-hematopoietic cells? Understanding these fundamental questions would give an important contribution to the definition of the basic mechanisms of innate immunity and to the comprehension of the link between the immune and the endocrine systems.

### Conflict of interest statement

The authors declare that the research was conducted in the absence of any commercial or financial relationships that could be construed as a potential conflict of interest.
